# 

**Published:** 1887-12-03

**Authors:** 


CONTENTS.

**London's Problem Solved** - - - - - 159

**The Doctor's Pulpit.**—Patients and Doctors - - 161

**Modern Drug Laboratories.** By our SPECIAL COMMISSIONER - - - - - - 162

**Homes in the Hills for Nursing Sisters.** By THE LADY NORA ROBERTS - - - - - - 163

**Dairies Old and New** - - - - - - 164

**Clinical and Therapeutic Notes.** By C. R. ILLINGWORTH, M.D., M.R.C.S. - - - - - - 165

**Notes and News** - - - - - - - 166

**A Proposed British Nnrsing Association** - - 168

**Annotations.**—A Great Opportunity.—An Indelible Dishonour. —Are we a Capable People? - - - - - 169

**Amusements with Profit for all Ages** - - - 170

**East and West.** By LESLIE KEITH. Chapter X. - - 171

**The Case of Miss Buchanan** - - - - - 172

**A Noble Profession: Nursing the Sick.**—The Nursing Sisters of St. Mary, New Kent Road. By a MEMBER of the SISTERHOOD - - - - - - -

**Hints for Probationer Nurses** . - - - -

